# Supplementary material for: The genome sequence of the Common Spotted Hoverfly, Eupeodes luniger (Meigen, 1822)
Source: Wellcome Open Res. 2023 Oct 18;8:472. [Version 1] doi: 10.12688/wellcomeopenres.20144.1 (PMC11128045; doi:10.12688/wellcomeopenres.20144.1)
Supplement: Peer review history

# Reviewer report: Reviewer response for version 1

**DOI:** 10.21956/wellcomeopenres.22306.r81595

**Published:** 2024-05-28

**Authors:** Adam James Reid

**Affiliations:** 1 University of Cambridge, Cambridge, UK

Hawkes *et al.* present a very high-quality genome assembly for the Common Spotted Hoverfly. The data and manuscript are well presented. The methods used are state-of-the-art.

 Major comments: none

 Minor comments: none

 While the particular organism and its genome sequence are interesting in their own right, the continued generation of high quality genomes by the Darwin Tree of Life project team will underpin a great deal of future work on evolutionary biology.

Are sufficient details of methods and materials provided to allow replication by others?

Yes

Is the rationale for creating the dataset(s) clearly described?

Yes

Are the datasets clearly presented in a useable and accessible format?

Yes

Are the protocols appropriate and is the work technically sound?

Yes

Reviewer Expertise:

Computational biology, genomics, transcriptomics, parasitology, developmental biology

I confirm that I have read this submission and believe that I have an appropriate level of expertise to confirm that it is of an acceptable scientific standard.

# Reviewer report: Reviewer response for version 1

**DOI:** 10.21956/wellcomeopenres.22306.r76497

**Published:** 2024-05-25

**Authors:** Lucio Navarro-Escalante

**Affiliations:** 1 Department of Molecular Biosciences, The University of Texas at Austin, Austin, Texas, USA

**Overall**

 This paper reports the chromosomal-level genome sequences of the hoverfly *Eupeodes* *luniger*, a wasp-mimicking fly in the Syrphidae family. The genome was sequenced and assembled using PacBio long-reads and Hi-C Illumina reads. The mitochondrial genome is also reported here. This assembly represents a high quality full genome. In general the methods offer sufficient details and all raw data is publicly available. I support the indexing of this report and its data.

 Minor comment:

 When describing the use of MitoHiFi in the methods, authors should clearly indicate whether MitoFinder or MITOS was used for mitochondrial genome assembly.

Are sufficient details of methods and materials provided to allow replication by others?

Partly

Is the rationale for creating the dataset(s) clearly described?

Yes

Are the datasets clearly presented in a useable and accessible format?

Yes

Are the protocols appropriate and is the work technically sound?

Yes

Reviewer Expertise:

Insect genomics, insect-microbe symbiosis, symbiont engineering

I confirm that I have read this submission and believe that I have an appropriate level of expertise to confirm that it is of an acceptable scientific standard.

# Reviewer report: Reviewer response for version 1

**DOI:** 10.21956/wellcomeopenres.22306.r76505

**Published:** 2024-04-08

**Authors:** De-Long Guan

**Affiliations:** 1 Shaanxi Normal University, Xi'an, Shaanxi, China

Dear Editor

 I have completed my review of the manuscript and I am pleased to report that I found no significant issues requiring further attention. The article is well-crafted, with a clear and concise presentation of the research topic, robust methodology, and sound analysis. The results are well-supported and the discussion is insightful, providing valuable contributions to the field.

 **Major Points:**

 1. **Scientific Significance:** The publication is a notable achievement as it presents the inaugural genome sequence for Eupeodes luniger, enriching our understanding of this species' biology and ecology. The genome sequence opens doors to investigate evolutionary patterns, genetic diversity, and the functional aspects of the hoverfly's genetics.

 2. **Genome Assembly Quality:** The quality of the genome assembly is commendable, with a substantial contiguity. The meticulous use of Hi-C data for scaffolding, along with manual curation, demonstrates a high level of precision and attention to detail in the assembly process.

 3. **Ecological and Agricultural Relevance:** The hoverfly's ecological role as a pollinator and its larvae's pest-control function by consuming aphids are well-documented. The genome sequence can serve as a foundation for research aimed at developing integrated pest management strategies, potentially revolutionizing sustainable agricultural practices.

 4. **Methodological Advancements:** The article exemplifies the application of state-of-the-art sequencing technologies, such as Pacific Biosciences single-molecule HiFi long reads and Hi-C for chromosomal conformation capture. These techniques represent the forefront of genomic research and offer a blueprint for future genomic studies on a variety of species.

 **Minor Points:**

 1. **Peer Review Status:** The article, at the time of its initial publication, was awaiting peer review. This indicates that while the research is presented by respected institutions, it has not yet been vetted by external experts in the field. This is a critical step in the scientific process that will ultimately strengthen the credibility of the findings.

 2. **Potential for Further Analysis:** The genome assembly lays the groundwork for in-depth functional annotation, which could reveal key genes and their roles in the hoverfly's biology. Future research could focus on annotating the genome to enhance our understanding of the species' unique characteristics and evolutionary adaptations.

 3. **Comparative Genomics:** The genome sequence offers a valuable resource for comparative genomics studies with related species. Such comparisons could shed light on the genetic underpinnings of the hoverfly's distinctive traits and its adaptive evolutionary history. This represents an area ripe for further exploration.

 I believe the article on the Common Spotted Hoverfly's genome sequence provides an invaluable resource for future research endeavors and has the potential to inform agricultural practices and conservation strategies.

Are sufficient details of methods and materials provided to allow replication by others?

Yes

Is the rationale for creating the dataset(s) clearly described?

Yes

Are the datasets clearly presented in a useable and accessible format?

Yes

Are the protocols appropriate and is the work technically sound?

Yes

Reviewer Expertise:

Insect genomics,comparative genomics,bioinformatics, Evolutionary Biology, Acrididae, Tetrigidae, and Papilionidae.

I confirm that I have read this submission and believe that I have an appropriate level of expertise to confirm that it is of an acceptable scientific standard.
